# Epithelial cells sense local stiffness via Piezo1 mediated cytoskeletal reorganization

**DOI:** 10.3389/fcell.2023.1198109

**Published:** 2023-05-24

**Authors:** Deekshitha Jetta, Tasnim Shireen, Susan Z. Hua

**Affiliations:** ^1^ Department of Mechanical and Aerospace Engineering, University at Buffalo, Buffalo, NY, United States; ^2^ Department of Physiology and Biophysics, University at Buffalo, Buffalo, NY, United States

**Keywords:** Piezo1 channels, ECM stiffness, mechanobiology, MDCK cells, focal adhesions, epithelial remolding

## Abstract

Local substrate stiffness is one of the major mechanical inputs for tissue organization during its development and remodeling. It is widely recognized that adherent cells use transmembrane proteins (integrins) at focal adhesions to translate ECM mechanical cues into intracellular bioprocess. Here we show that epithelial cells respond to substrate stiffening primarily via actin cytoskeleton organization, that requires activation of mechanosensitive Piezo1 channels. Piezo1 Knockdown cells eliminated the actin stress fibers that formed on stiff substrates, while it had minimal effect on cell morphology and spreading area. Inhibition of Piezo1 channels with GsMTx4 also significantly reduced stiffness-induced F-actin reorganization, suggesting Piezo1 mediated cation current plays a role. Activation of Piezo1 channels with specific agonist (Yoda1) resulted in thickening of F-actin fibers and enlargement of FAs on stiffer substrates, whereas it did not affect the formation of nascent FAs that facilitate spreading on the soft substrates. These results demonstrate that Piezo1 functions as a force sensor that couples with actin cytoskeleton to distinguish the substrate stiffness and facilitate epithelial adaptive remodeling.

## Introduction

Epithelial cells respond to substrate mechanical signals with a continuous remodeling process that maintains epithelial barrier integrity. Hardening of the substrate occurs during changes in physiological states such as aging or due to development of disease states including tumors (Wells, 2013; Semenza, 2016). The variation in local mechanical properties influences many cell behaviors, which alters cell proliferation, differentiation and migration (Pelham and Wang, 1997). The mechanical inputs at the substate is commonly thought to be transmitted via adhesion proteins such as integrins (Ingber, 2003; Schwartz, 2010). Mechanosensitive Piezo1 protein is found in locations side-by-side with integrins and is sensitive to substrate mechanical properties, suggesting Piezo1 and integrins may collaborate to regulate cell-ECM interactions (Coste et al., 2010; Coste et al., 2012; Segel et al., 2019).

Tissue cells adapt to changes in local mechanical environments through various structural and morphological rearrangements. Fibroblasts grown on stiffer substrates generated more traction force and exhibited a larger and flatter morphology compared with soft substrates (Lo et al., 2000; Yeung et al., 2005). Epithelial cells develop abundance of actin stress fibers on hard substrates, that are associated with larger cell expansion, disruption of cell-cell junctions, and upregulation of proliferation (Kim and Asthagiri, 2011; Verma et al., 2012). In contrast, these cells on soft substrates grow into a tightly connected cell sheet with strong adhesion protein expression (E-cadherin and ZO-1) along the cell periphery, that overlaps with peripheral F-actin (Kim and Asthagiri, 2011; Balcioglu et al., 2020). Mechanical properties of substrates also markedly influence cell migration and cells exhibit higher mobility and move velocity on stiffer substrates (Pathak and Kumar, 2012; Nasrollahi et al., 2017).

Piezo1 channels are sensitive to a range of substrate mechanical properties including confinements via micropatterns and local ECM hardening (Segel et al., 2019; Emig et al., 2021; Jetta et al., 2021). In the brain, substrate stiffness alters the ratio of neurons to astrocytes during neural stem cell differentiation via Piezo1 activities (Pathak et al., 2014). A recent study discovered that age-related substrate stiffening changed the behavior of oligodendrocyte progenitor cells, resulting in a decline in tissue regeneration (Segel et al., 2019). Inhibiting Piezo1 superseded the hardening effects and recovered the functional activity of aged cells (Segel et al., 2019). In epithelia, Piezo1 was overexpressed at the cell edges of sparse regions, which triggered rapid cell division promoting epithelial confluence (Gudipaty et al., 2017). In brain tumor cells, tissue stiffening promoted Piezo1 overexpression, that increased glioma aggression through Piezo1 mediated ion conductance (Chen et al., 2018).

Substrate stiffening triggers Piezo1 mediated Ca^2+^ influx, that modulates Piezo1 activity enhances actin polymerization in cells (Atcha et al., 2021). It has been reported that traction forces alone could activate Piezo1 to generate local Ca^2+^ flickers in neural stem cells (Ellefsen et al., 2019). Piezo1 mediated Ca^2+^ influx promotes RhoA/ROCK actomyosin contractility critical for F-actin assembly and myotube formation (Tsuchiya et al., 2018). Piezo1 mediated Ca^2+^ influx at focal adhesions has been shown to trigger various Ca^2+^-dependent signaling pathways, altering cell morphology, reorganization, and migration (McHugh et al., 2010; Chen et al., 2018). Piezo1 may also have effects beyond its role as an ion channel. Cells become stiffer when grown on hard substrate, which required an increase in Piezo1 expression level but not ion influx (Emig et al., 2021).

In this study, we examined remodeling of MDCK cells on a range of substratum stiffness. The role of Piezo1 is studied by knockdown of Piezo1 with miRNA and by inhibiting Piezo1 activity with specific pharmacological inhibitor GsMTx4. We show that Piezo1 is involved in the formation of thick actin stress fibers that together with mature FAs facilitate cell expansion on hard substrates, while it has subtle effect on cell spreading that only involves nascent FAs on soft substrates. Knockdown of Piezo1 with miRNA or inhibiting Piezo1 channels resulted in significant reduction in F-actin intensity in the cell interior, so that cells lost the distinction of hard and soft substrates. The results suggest that Piezo1 functions as a sensor for stiffness and actin cytoskeleton is part of this sensing apparatus.

## Materials and methods

### Modification of substrate stiffness

The PDMS substrates of varying stiffness were made from Sylgard 184 and Sylgard 527 (Dow Corning). The stiffness of the substrates was modified by varying ratios of elastomer base to curing agent targeting three regions, hard: ∼1 MPa, medium: ∼50 kPa (Balaban et al., 2001), soft: <5 kPa (Moraes et al., 2015). Glass coverslips (∼1 GPa) were used for comparison (Supplementary Table S1). For the experiments, two-well (3 mm diameter) chips were made by bonding a PDMS compartment on coverslip. PDMS mixtures were placed into the wells and cured for 24 h at room temperature. To separate the effect of surface chemical variation from mechanical properties, all substrates were treated with fibronectin (50 μg/ml) in phosphate-buffered saline (PBS) and incubated for 45 min prior to experiments.

### Cell morphology evaluation

The spreading area of each cell was quantified using ImageJ (NIH) with MATLAB plugin. Briefly, brightfield images were imported to MATLAB and converted to binary images, and individual cells were identified using built-in “adaptive threshold.” The images were processed with several erosions and dilations to clearly define the cell boundary. The binary images were then transferred back to ImageJ and the cell areas were measured. F-actin thickness was measured using Plot Profile in ImageJ. Focal adhesions were selected manually and analyzed using ImageJ. Multiple cells were selected from each image and multiple images were used. A minimum of 4 experiments were performed under each condition.

### Cell culture and transfection

Madin-Darby Canine Kidney (MDCK) cells (ATCC) were grown to confluence in Dulbecco’s Modified Eagle Medium (DMEM) containing 10% fetal bovine serum, 1% penicillin and streptomycin. The cells were trypsinized and seeded in the wells and allowing to grow for 30 min in the incubator. The cells were then washed with media that removed unattached cells and grown for additional 2 h. Live cell imaging were conducted in a stage-top incubator (INUB-ZILCSD-F1-LU, Tokai Hit Co., Ltd., Japan), maintained at 37°C and 5% CO_2_. No-phenol Red DMEM media with 10% fetal bovine serum (Gibco, TX) was used for fluorescence imaging to reduce the background illumination. Isotonic saline solution was used for experiments with GsMTx4 and Gd^3+^, as it was known that culture media reduced the efficiency of the peptide (Bae et al., 2011).

Piezo1 knockdown was performed using previously validated Piezo1 miRNA targeting Piezo1 and co-expressed with EGFP to verify transfection (Jetta et al., 2019). Cells were cultured to ∼60% confluence and transfected with plasmid DNA miRNA (0.4 µg) using Effectene (Qiagen, Valencia, CA) at 1:50 DNA to effectene ratio. Cells were incubated in the transfection media for another 48 h prior to experiments. The transfection efficiency was ∼25%.

### Immunostaining

Cells were fixed with 4% paraformaldehyde for 15 min, and permeabilized with Triton X-100 (0.1% in PBS) for 15 min, followed by blocking with 5% Goat Serum (Sigma-Aldrich) for 1 h at room temperature. For staining paxillin, blocking buffer, made with 5% Goat Serum and 0.01% Triton in PBS was used for diluting primary and secondary antibodies. Cells were incubated with primary antibody, Anti-Paxillin (BDB610052, Fischer Scientific), at 1:50 dilution overnight at 4°C and secondary antibody, Alexa fluor 488 (A11001-Thermo Fischer) at 1:200 for 1 h at room temperature. F-actin was stained with Phalloidin Alexa Flour 568 (A12380, Fischer Scientific) at 1:100 in PBS and was incubated at room temperature for 1 h. After every step, cells were washed three times with PBS. Imaging was done in slow fade Gold Anti-fade Reagent (1:100, Invitrogen) in PBS to protect from photobleaching. Images were acquired using an inverted microscope (Axiovert 200M, Zeiss) with a CCD camera (AxioCam, Zeiss). Fluorescence images were obtained with two filter sets: (Ex: 470/40 nm; Em: 525/50 nm) and (Ex: 550/25, Em: 605/70), and a ×63 oil immersion objective. The B/W images were obtained using a ×20 objective.

### Ca^2+^ measurements

Cytosolic Ca^2+^ was measured using Ca^2+^ sensitive dye, Fluo-4 AM (5 μM, Invitrogen). The normalized Ca^2+^ intensity was calculated using $\frac{∆F}{F_{0}}=\frac{F-F_{0}}{F_{0}}$ , where *F* and *F*_*0*_ are the mean fluorescence intensities of individual cells at time t and t = 0, respectively.

### Solution and chemicals

GsMTx4 was purified and diluted in saline to a final concentration of 5 μM. Yoda1 (Tocris Bioscience) was dissolved in DMSO as stock solution (48 mM), then diluted in saline to a final concentration of 25 µM. Gadolinium chloride (Sigma-Aldrich, St. Louis, MO) was freshly prepared and diluted to 20 µM in saline.

### Statistical analysis

The data is shown as the mean ± standard error of the means (s.e.m.). Statistical analysis was performed using two sample *t*-test, and value of *p* < 0.05 was considered statistically significant.

## Results

### Substrate stiffness primarily modulates actin cytoskeleton

**FIGURE 1:**
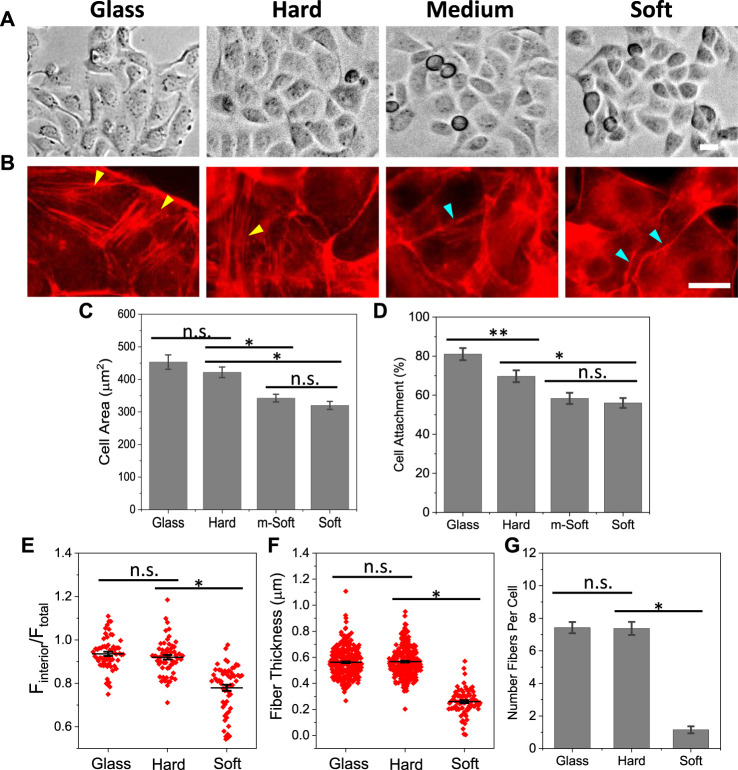
Effect of substrate stiffness on cell spreading and F-actin organization. **(A)**: MDCK cells grown on substrates of varying stiffness for 2.5 h. **(B)** Immunostaining cells with phalloidin showed that cells form thick stress fibers on glass and hard substrates, and minimal stress fibers on soft substrate (yellow arrows indicate stress fibers, blue arrows indicate peripheral actin rings). **(C)** Statistical analysis of spreading area, showing a slight reduction on soft substrate (*n* = 90 from more than 4 experiments for each condition, *p < 0.001). **(D)** Percentage of attached cells on different substrates (*n* = 30 frames, *p < 0.001, **p < 0.05). **(E)** Mean fluorescence intensity of F-actin from cell interior normalized with whole cell. It shows the actin intensity on stiffer substrate is significantly higher than that on soft substrate (*n* = 60, *p < 0.001). **(F)** F-actin thickness on different substrates, showing the fibers are significantly thicker on stiffer substrates (*n* = 300 for glass and hard, 80 for soft, measured from 30 cells in each condition, *p < 0.001). **(G)** Number of actin fibers per cell (*n* = 30 cells, *p < 0.001). Scale bars represent 20 µm.

To investigate the effect of local mechanical properties, we cultured MDCK cells on four different substrates, glass, hard, medium-soft and soft with stiffness ranging from ∼1 GPa to ∼5 Pa (Supplementary Table S1). Cells spread to a full extent on all substrates within 3 h, and showed hexagonal/polygonal shape, typical for epithelial cells (Figure 1A). The spreading area of individual cells varies slightly depending on the substrate stiffness. Cells spread to larger area (∼20%) on glass and hard substrates compared with the soft substrates (Figures 1A, C). However, the organization and amount of actin fibers vary drastically on different substrates. On stiffer substrates, glass and hard, cells developed high density actin stress fibers across the cell body that extends to cell protrusions (yellow arrows, Figure 1B). On soft substrates, the actin fibers arranged along the cell periphery to form a ring-shape boundary and there was no stress fiber like structure in the cell interior (blue arrows, Figure 1B). The amount of F-actin was quantified by the mean fluorescence intensity of cell interior and normalized with the mean intensity of whole cell, showing that interior F-actin intensity is greater for the hard substrates compared with soft ones (Figure 1E). The actin fibers were quantified by the thickness and number of fibers in individual cells, which shows that both thickness and number of fibers are significantly greater on stiffer substrates (Figures 1F, G, *p < 0.001). The percentage of attached cells was lower on soft substrates (Figure 1D, *p < 0.001), and only attached cells were used for analysis. This result indicates actin cytoskeleton are sensitive and responsive to the stiffness of the substrates.

### Piezo1 is essential for detection of substrate stiffness

**FIGURE 2:**
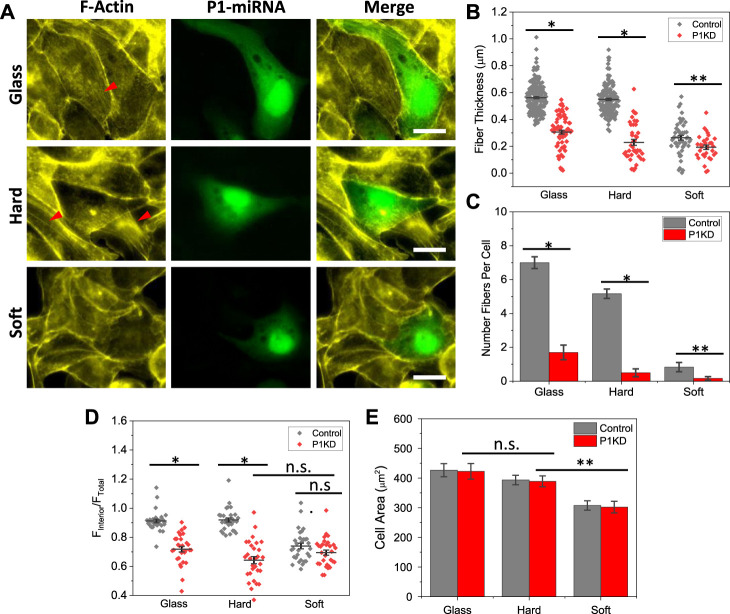
Reduction of F-actin intensity in Piezo1 Knockdown cells. **(A)** Images of F-actin staining in Piezo1 knockdown (P1KD) cells on glass, hard and soft substrates. P1KD cells co-expressing GFP are compared with control cells in the same culture well, showing F-actin intensity was significantly lower in P1KD cells (red arrows indicate F-actin). **(B)** Actin fiber thickness in P1KD were compared with control cells, and show that Piezo1 knockdown reduced fiber thickness significantly (*n* = 190/70, 150/40, 50/30 for control/P1KD on glass, hard and soft, respectively, from 30 cells in each condition, *p < 0.001, **p < 0.05). **(C)** Number of fibers per cell (*n* = 30, *p < 0.001, **p < 0.05). **(D)** Mean actin fluorescence intensity in the cell interior in P1KD cells is lower than control cells on glass and hard substrates, but the reduction is insignificant for soft substance (*n* = 30 for all conditions, *p < 0.001). **(E)** Statistical analysis shows that P1KD cells extended to similar sizes, and Piezo1 knockdown had no significant effect on cell spreading (*n* = 50 for glass and hard, *n* = 40 for soft, **p = 0.002). Scale bars represent 20 µm.

MDCK cells endogenously express mechanosensitive Piezo1, that can be activated by changes in membrane tension or fluid shear stress (Gudipaty et al., 2017; Jetta et al., 2019). To assess whether Piezo1 is responsible for detection of substrate stiffness, we knockdown Piezo1 with miRNA, and observed the cell shape and actin reorganization with varying substrate stiffness. Cells were transfected with Piezo1 miRNA and co-expressed with EGFP to verify transfection (green, SM Supplementary Figure S1). Piezo1 knockdown (P1KD) cells showed significantly low intensity of actin fibers in the cell interior on all substrates (Figure 2A). These cells developed F-actin rings along the cell periphery on hard and glass substrates. In contrast, the nearby control cells (non-transfected), showed strong actin stress fibers (red arrows, Figure 2A). Piezo1 knockdown appeared to have subtle effect on cells on soft substrates (Figure 2A). The thickness of actin fibers in P1KD cells was measured and compared with the control cells, which shows Piezo1 knockdown reduced the fiber thickness significantly on stiffer substrates (Figure 2B, *p < 0.001, **p < 0.05). The stress fibers in P1KD cells were diminished (Figure 2C, *p < 0.001, **p < 0.05). The F-actin intensity in interior of the P1KD cells is also reduced significantly (Figure 2D, *n* = 30 for all, *p < 0.001). However, the cell shape was not altered, nor the spreading area (Figure 2E, *n* = 50 for glass and hard, *n* = 40 for soft, **p < 0.05). This result indicates that Piezo1 along with actin cytoskeleton forms a vital force transduction apparatus in epithelial cells.

### Inhibition of Piezo1 current eliminates cytoskeleton response

**FIGURE 3:**
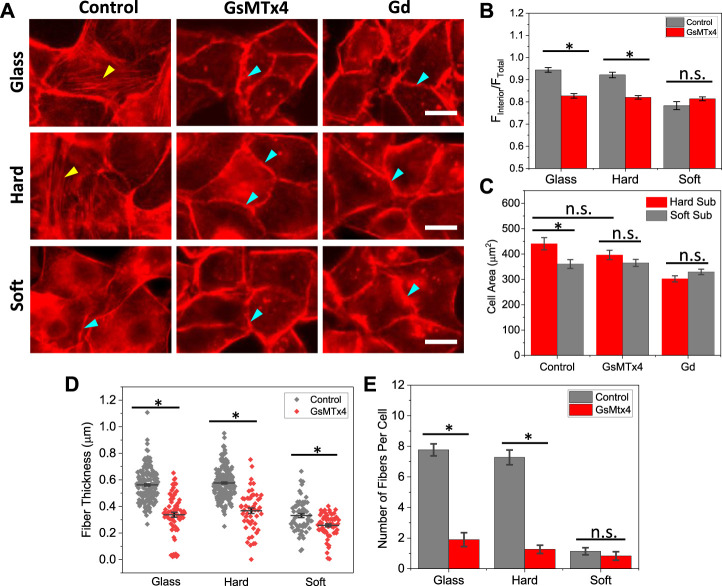
Effect of Piezo1 inhibitors on F-actin organization. **(A)** Images of F-actin reorganization in the presence of GsMTx4 and Gd^3+^, showing the stress fibers disassembled in the presence of inhibitors (yellow arrows indicate stress fibers, blue arrows indicate peripheral actin rings). The inhibitors were added during cell seeding, and the cells were fixed and stained after 2.5 h. Scale bars represent 20 µm. **(B)** Statistical analysis of interior actin intensity with and without GsMTx4, which shows that blocking Piezo1 channels reduced the actin intensity on stiffer substrates (*n* = 50 for all conditions, *p < 0.001). **(C)** Comparison of spreading areas on hard and soft substrates in the presence of inhibitors, showing that blocking Piezo1 channels did not drastically alter cell spreading (*n* = 50 for all conditions, *p < 0.005). **(D)** F-actin thickness with and without inhibitor, showing that blocking Piezo1 channels reduced the thickness of fibers significantly (*n* = 170/70; 220/50; 60/60 for control/drug on glass, hard, soft, respectively, measured from 30 cells in each condition, *p < 0.001). **(E)** Number of actin fibers per cell (*n* = 30 cells, *p < 0.001).

To assess if Piezo1 mediated cation current is needed for cell remodeling on hard substrates, we blocked the channels using Piezo1 specific inhibitor, GsMTx4. On hard substrates, inhibiting Piezo1 significantly reduced the actin stress fibers in the cell interior, but it has little effect (<10%) on cell spreading (Figures 3A, C). Some F-actin appeared in punctuated form in the cell interior after treatment. On soft substrates, neither actin organization nor the cell area were altered by Piezo1 inhibition (Figures 3A, C). We quantified the thickness and number of actin fibers in cells and found that inhibiting Piezo1 channels reduced the fiber thickness and the number of stress fibers significantly (Figures 3D, E, *p < 0.001). As controls, we used non-specific mechanosensitive channel (MSC) inhibitor Gd^3+^ to inhibit all MSCs. The interior actin stress fibers were eliminated in all cells. The cell spreading area was also moderately reduced in the presence of Gd^3+^, it is because some cells started to shrink under the treatment of the drug for 2.5 h. Thus, Piezo1 mediated Ca^2+^ current is necessary in stiffness triggered F-actin reorganization. We have previously shown that cell expansion requires opening of Piezo1 channels with Ca^2+^ influx that leads to activation of Rho-ROCK pathway (Jetta et al., 2021). This result is consistent with previous findings, and further shows that Piezo1 mediated Ca^2+^ signal is involved in cell sensing of stiffness.

### Piezo1 activation enhances actin fibers and FAs

**FIGURE 4:**
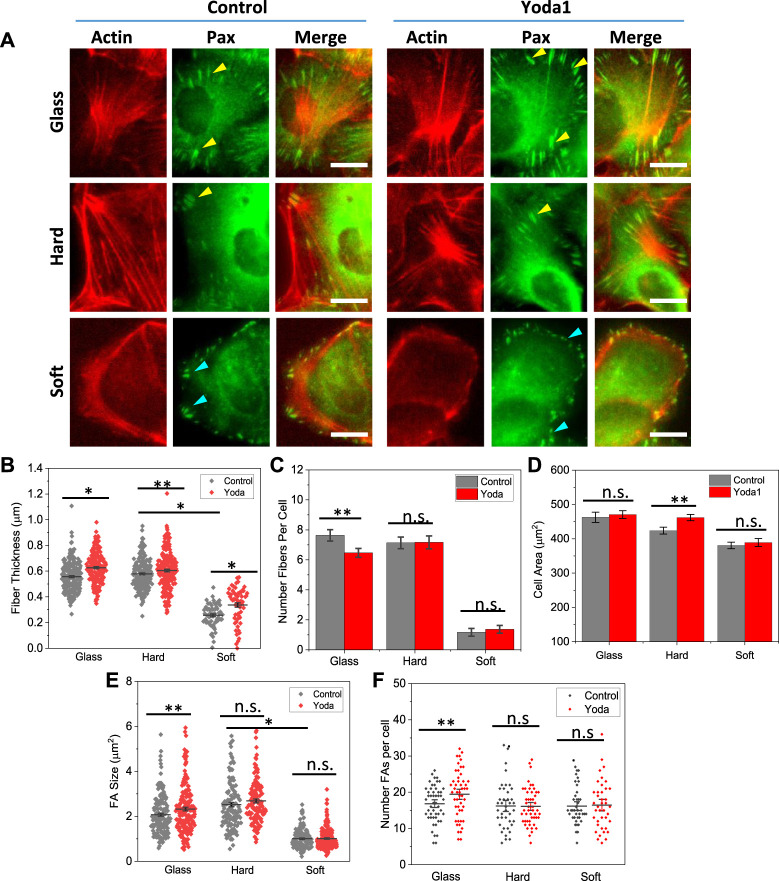
Role of Piezo1 in focal adhesion development on various substrates. **(A)** Merged images of actin (RFP) and paxillin (GFP) in control and Yoda1 treated MDCK cells. The drug was added at 60 min after cell seeding and the cells were fixed at 2.5 h. It shows treatment of Piezo1 agonist Yoda1 enhanced the stress fibers on glass and hard substrates, but had no effect on the soft substrate. **(B)** Effect of Yoda1 on fiber thickness, showing that the drug treatment enhanced the fiber thickness on all substrates (*n* = 230/190, 60/60, 210/210 for control/Yoda1 on glass, hard, soft, respectively, from 30 cells in each condition, *p < 0.001, **p < 0.05). **(C)** Yoda1 treatment did not affect the number of fibers in cells (*n* = 30 cells, **p < 0.05). **(D)** Yoda1 treatment caused only small increase in cell spreading area (*n* = 100 for all conditions, **p = 0.005). **(E)** Effect of Yoda1 on FA size, showing an increase in size on glass, but negligible effect on hard and soft substrates (*n* = 160/180, 125/130, 180/210, for control/Yoda1 on glass, hard, soft, respectively, from 30 cells in each condition, **p < 0.05). **(F)** Effect of Yoda1 treatment on number of FAs (*n* = 30 cells, *p < 0.05). Scale bars represent 10 µm.

We evaluated the effect of stiffness on focal adhesions by immunostaining Paxillin, a protein located in the focal adhesion complex. We found that FA enlargement was strictly modified by the stiffness. Cells developed large mature FAs on stiffer substrates, that were aligned with stress fibers at the ends (yellow arrows, Figure 4A, Control panel). Some short FAs also exist, however, those FAs were located in the middle of the cell or dissociated with the stress fibers (not shown). The FAs are also present in Piezo1 knockdown cells, although the size of the FAs is much smaller (Supplementary Figure S2). P1KD only reduced the number of FAs slightly (Supplementary Figure S2). On soft substrates, the FAs were much smaller and shorter compared with those on hard substrates, and they were distributed along the cell periphery. Interestingly, these FAs are mostly radial although they are not associated with stress fibers (blue arrows, Figure 4A). We measured the size of FAs and found that the mean FA size on glass and hard substrates are significantly larger than that on soft substrate (Figure 4E, *p < 0.001). The stiffness had minimal effect on the number of FAs in individual cells (Figure 4F). These results show that cell spreading was facilitated by two stage of FAs, mature FAs that couple with stress fibers to transmit traction forces, and nascent FAs that are linked to actin mesh providing guidance for spreading. Small nascent FAs located at the lamellipodium edge can facilitate cell spreading without stress fibers facilitated traction forces.

We then evaluated the role of Piezo1 on F-actin and FAs with Piezo1 agonist, Yoda1. Yoda1 was added to the culture media at 1 h after seeding the cells, and cells were fixed and co-stained at 2.5 h. Yoda1 treated cells showed thicker actin fibers and larger mature FAs compared with control cells, while the organization of actin and FAs remain the same (Figure 4A). Statistical analysis shows that fiber thickness increased on all substrates after Yoda1 treatment (Figure 4B, *p < 0.001, **p < 0.05), while the number of fibers did not show significant change (Figure 4C). The enhancement of actin fibers and FAs did not result in a significant increase in cell area (Figure 4D).

FA sizes increased moderately on stiffer substrates after Yoda1 treatment (Figure 4E). On soft substrates, the effect of Yoda1 on size and number of FAs per cell is negligible (Figure 4F). The existence of nascent FAs at cell protrusions provided the guidance for cells expansion. These FAs were never grown to mature even in the fully spreading cells on soft substrate.

## Discussion

Epithelial cells can detect and continuously adapt to substrate mechanical environments. In this study, we have shown that mechanosensitive Piezo1 is coupled with actin cytoskeleton to detect the stiffness and facilitate the adaptive response in MDCK cells. These cells form abundant thick parallel actin bundles on stiffer substrates, which reorganize to a peripheral actin ring on soft substrates. Piezo1 knockdown cells lost their differentiation of stiffness on different substrates and showed a uniform peripheral actin structure. Inhibition of Piezo1 channels with specific inhibitor significantly reduced the cell response. This indicates that Piezo1 mediated cation currents, possibly Ca^2+^ influx, plays an essential role. Activation of Piezo1 with specific agonist (Yoda1) enhanced the thick F-actin fibers and maturation of FAs on stiffer substrates, while having subtle effect in cell spreading areas. Thus, Piezo1 and actin cytoskeleton are essential force transduction elements in this sensing apparatus.

It is well known that stiffness of extracellular substrates can modulate cell adhesion, spreading, and division. Previous studies have shown that cells grown on stiffer substrates spread to larger area, while cells on soft substrates spread to a lesser extent (Discher et al., 2005; Geiger et al., 2009). However, the stiffness has shown a minimal effect on the spreading area in MDCK cells, the effect is mainly on the deployment of actin organization and associated FA distribution (Figure 1). Cells developed high density parallel stress fibers on stiffer substrates while they have peripheral F-actin rings on soft substrates (Figure 1). Cells also exhibited mature FAs on stiffer substrates, that are further enhanced by Piezo1 agonist (Figure 4). Knockdown of Piezo1 results in disassembly of actin bundles in the cell interior and nascent FAs (Figure 2; Supplementary Figure S2). The cells thus, lost their sensitivity to local stiffness. The role of Piezo1 in facilitating extracellular matrix mechanics has been observed in various cells. In human atrial fibroblasts, cell stiffness increased when grown on stiffer substrate, that required increase in Piezo1 expression level but not ion influx (Emig et al., 2021). Increase in Piezo1 expression level led to the formation of thick actin bundles increasing the stiffness of human atrial fibroblasts (Emig et al., 2021). In macrophages, substrate stiffening triggers Piezo1 mediated Ca^2+^ influx, that promotes actin polymerization and modulates macrophage activation (Atcha et al., 2021). In human foreskin fibroblast cells, Yao et al. (2022) has shown that Piezo1 is required for FA maturation, and these proteins colocalize with FAs and enhance their maturation in a force-dependent manner. This is consistent with our observations in MDCK cells, where cells developed mature FAs only on hard substrates and Piezo1 agonist promoted FA growth (Figure 4).

Piezo1s can be activated by changes in membrane tension to transport Ca^2+^ (Gudipaty et al., 2017), and they may also functions besides acting as a cation channel, which involves direct interactions with integrin subunits to modulate cell adhesions (Emig et al., 2021; Jetta et al., 2021). Piezo1 proteins have multiple locations in MDCK cells, from the nuclear envelope to the cell membranes, and they can translocate following a tension gradient (Gudipaty et al., 2017). Our results show that agonist incited Ca^2+^ level was much higher on the hard substrates compared with soft substrates (Supplementary Figure S3), indicating that more Piezo1s were located on the cell membranes on stiffer substrates. Inhibiting Piezo1 current with specific inhibitor GsMTx4 considerably reduced the F-actin fluorescence intensity on stiffer substrates (Figure 3). Conversely, activation of Piezo1 with agonist Yoda1 significantly enhanced the intensity of thick actin fibers (Figure 4), indicating that Piezo1 mediated Ca^2+^ influx is involved. Increasing substrate stiffness resulted in an increase in traction forces in adherent cells (Riveline et al., 2001; Byfield et al., 2009; Califano and Reinhart-King, 2010). Piezo1s can be activated by myosin II mediated traction force to generate local Ca^2+^ flickers at focal adhesions (Ellefsen et al., 2019). The Piezo1 mediated Ca^2+^ influx, in turn, promotes RhoA/ROCK actomyosin activities critical for F-actin disassembly and organization through a positive feedback loop (Tsuchiya et al., 2018; Atcha et al., 2021). We and others have previously shown that inhibiting the Rho-ROCK pathway reversibly inhibited F-actin assembly in MDCK cells (Ye et al., 2014; Jetta et al., 2021). Therefore, Piezo1 sensing function is achieved via Ca^2+^ dependent Myosin-II activated actin stress fiber polymerization in epithelial cells.

On the soft substrates cells exhibited a significant reorganization of F-actin, however, their spreading areas were only slightly reduced. Interestingly, knockdown Piezo1 or inhibiting the channels’ current showed no effect on cell spreading area on various stiffness spanning a broad range (Figure 3). This result indicates that substantial increase in stress fibers and contractile forces may not be necessary for cell expansion in epithelial cells. Detailed examination of FAs on various substrates shows that cell spreading on soft substrates was facilitated by the small nascent FAs, that never reached a mature size during cell expansion (Figure 4). These nascent FAs exist even in Piezo1 knockdown cells (Supplementary Figure S2). At low stiffness (less than 5 kPa), F-actin can exist in the form of a cortical mesh with no stress fibers (Solon et al., 2007). Cells exhibit low-density small FAs (Gerardo et al., 2019), and these FAs can grow with largely reduced myosin-II contraction forces (Oakes et al., 2012). A recent study showed that effective forces on a larger FA is reduced during its expansion, and nascent adhesions on soft substrate is an energetically favored state (Solowiej-Wedderburn and Dunlop, 2022). Thus, substantial forces arising from substrate stiffness regulate the F-actin assembly but may not be necessary for cell spreading. In conclusion, the mechanosensitive Piezo1 modulates F-actin assembly and arrangement, the coupling of Piezo1 and actin cytoskeleton provides a sensing apparatus for substrate stiffness.

## Data Availability

The original contributions presented in the study are included in the article/Supplementary Material, further inquiries can be directed to the corresponding author.
