# Supplementary material for: Epithelial cells sense local stiffness via Piezo1 mediated cytoskeletal reorganization
Source: Front Cell Dev Biol. 2023 May 24;11:1198109. doi: 10.3389/fcell.2023.1198109 (PMC10244755; doi:10.3389/fcell.2023.1198109)
Supplement: Supplementary file 1 [file Presentation1.pdf]

Supplemental Materials

Substrates	Materials (elastomer : curing agent)	Stiffness
**Glass**	Cover glass	> 1 GPa
**Hard**	S184 (10:1)	~1 MPa
**Medium soft**	S184 (40:1)	~ 50 kPa [Ref. Balaban, 2001]
**Soft**	S527 (1:0.8)	< 5 kPa [Ref. Moraes, 2015]

SM Table 1. Stiffness characterization of the substrates used. The modulus of glass and hard substrates were measured using AFM and the soft substrate were taken from the published data (Balaban et al., 2001; Moraes et al., 2015).

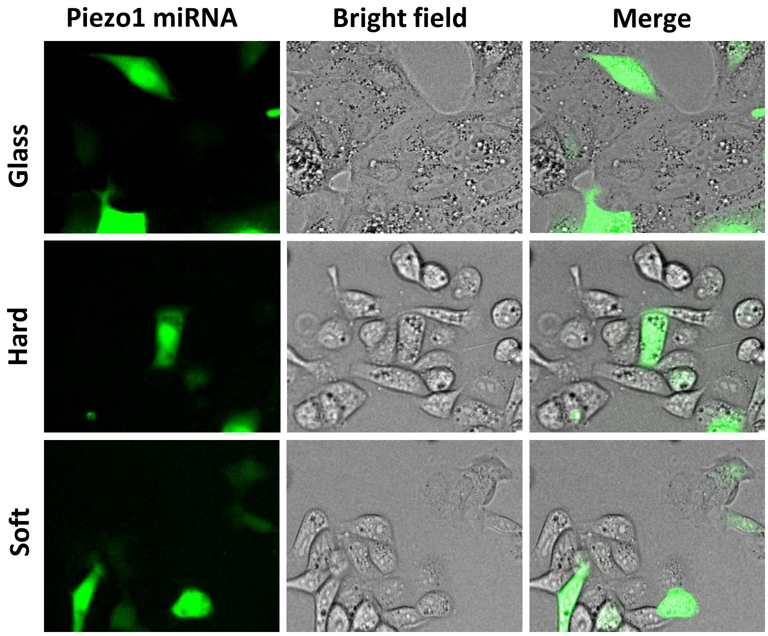

SM Figure 1. Characterization of Piezo1 knockdown in MDCK cells. *Piezo1* miRNA transfected cells were cultured on three substrates and allowed to spread for 2 hr. The P1KD cells were labeled by co-expressing GFP on the panels. The image shows knockdown of Piezo1 did not alter cell adhesion nor morphology. The transfection rate is ~ 25% on all substrates.

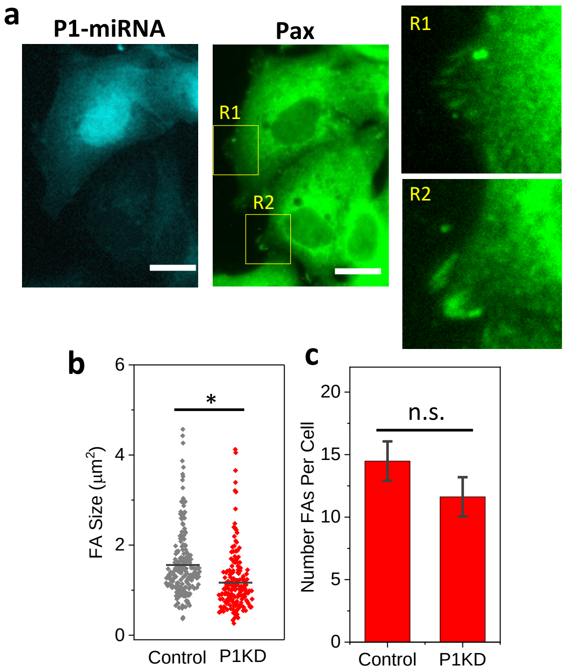

SM Figure 2. Characterization of focal adhesions in Piezo1 knockdown cells. MDCK cells were transfected with *Piezo1* miRNA and cultured on glass substrate, cells were fixed and immunestained with Paxillin after 2.5 hrs of seeding. **(a)** Image shows a transfected cell (P1KD, Cyan) and a neighbor non-transfected control cell, showing FAs exist in both control and P1KD cells. The sizes of FAs in P1KD cells (R1) are smaller than control cells (R2). **(b)** Statistical analysis of FA sizes shows that the difference between P1KD and controls is significant (n = 210 and 170 for control and P1KD, respectively, from 25 cells for each condition, $*p < 0.001$). **(c)** Piezo1 knockdown has minimal effect on number of FAs per cell (n = 25 cells). Scale bars indicate 20 μm.

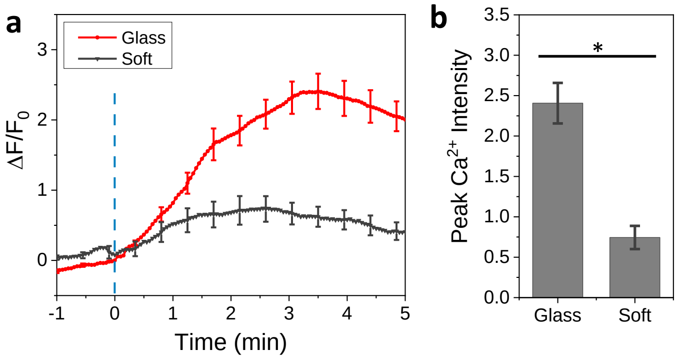

SM Figure 3. Stiffness dependent Ca^{2+} increase incited by Piezo1 agonist. (a) Time traces of Ca^{2+} changes in cells on glass and soft substrates. Yoda1 (50 μm) was added at time indicated by blue dashed line. Each curve was averaged over 30 cells from four experiments (n = 30). **(b)** Mean peak value for the two conditions (n = 30, $*p < 0.001$). It shows Piezo1 mediated Ca^{2+} uptake is greater on stiffer substrates.
